# Possible mechanisms of control of Fusarium wilt of cut chrysanthemum by *Phanerochaete chrysosporium* in continuous cropping fields: A case study

**DOI:** 10.1038/s41598-017-16125-7

**Published:** 2017-11-22

**Authors:** Ping Li, Jingchao Chen, Yi Li, Kun Zhang, Hailei Wang

**Affiliations:** 1 Henan Province Engineering Laboratory for Bioconversion Technology of Functional Microbes, College of Life Sciences, Henan Normal University, Xinxiang, 453007 China; 2 Advanced Environmental Biotechnology Center, Nanyang Environment and Water Research Institute, Nanyang Technological University, Singapore, 637141 Singapore

## Abstract

Continuous cropping is a universal challenge in agriculture because it has adverse physiological effects on plants, resulting in stunting, inferior quality, and even massive loss in harvest due to diseases. In this study, *Phanerochaete chrysosporium* was inoculated into the field in which cut chrysanthemum had already been continuously cropped for five years to control wilt disease. After 120 days of cultivation, the addition of *P*. *chrysosporium* significantly improved the physiological status of plants and changed the bacterial and fungal community structure in the soil. The bacterial quantity in the treatment increased by 1.76 times, but the fungal quantity, especially the quantity of *Fusarium oxysporum*, decreased significantly in comparison with the control. The investigation into the mechanisms of control of Fusarium wilt of cut chrysanthemum by *P*. *chrysosporium* showed that *P*. *chrysosporium* in soil can inhibit the growth of *F*. *oxysporum* and decrease *p*-hydroxybenzoic acid (HA), which stimulates the propagation of *F*. *oxysporum*. Based on current evidence, the inhibition by *P*. *chrysosporium* and change in HA appear to be the main causes of the alleviation of wilt disease in the treatment. Other factors, such as nutrients, might also have an influence on the wilt disease of cut chrysanthemum.

## Introduction

Cut chrysanthemum (*Chrysanthemum morifolium*), which is native to Asia and Northeastern Europe, is the oldest ornamental plant and a commercially important herb; thus, it has gained tremendous popularity around the world due to its wide range of biodiversity^1^. At present, the demand for cut chrysanthemum, the second most dominant cut flower in the market following roses, is soaring due to its extensive applications, such as in tea, medicine, ornaments, and food. Thus, the number of areas devoted to cultivation of cut chrysanthemum in China has grown significantly. In 2008, the quantity of national export reached 200 million, making the flower an important economic crop. However, during long periods of cultivation, the continuous monoculture has a negative impact on soil conditions, ultimately reducing chrysanthemum productivity. This phenomenon, generally referred to as the continuous cropping problem, is a universal challenge in agriculture and is observed in many agricultural systems, such as flowers, vegetables, grass, and fruit trees. The adverse effects caused by continuous monoculture include stunting, withering leaves, inferior quality, few flowers, and even massive loss in harvest due to diseases. For example, soil-borne diseases under continuous cropping conditions inhibit asparagus growth and severely decrease the yield and quality of soybean^2,3^.

The mechanism behind the adverse effects of continuous monoculture remains unclear. Hence, further research is necessary to find effective strategies to overcome the problem. Allelopathy is believed to be the main reason for problems associated with the continuous cropping of plants^4,5^. Autotoxicity, which is associated with allelopathy, has been extensively studied, and the root exudates of plants, especially phenolic acids, are believed to have a significant influence on both plant growth and soil characteristics^6,7^. Moreover, the continuous cropping mode can change the microbial community structure and cause the accumulation of pathogens and an increased incidence of various plant diseases^8,9^. For cut chrysanthemum, the main pathogen appearing in continuous cropping systems is *Fusarium oxysporum*: the wilt disease caused by this pathogen can irreversibly damage the growth of cut chrysanthemum.

Methods such as rotation, cultivation, and soil sterilization are used to control the wilt disease of cut chrysanthemum. The biological method is considered an environment-friendly approach and it has gradually replaced the application of traditional chemical pesticides to reduce pollution^10,11^. Microorganisms play a vital role in soil ecosystems, and they can serve as natural antagonists of plant pathogens^12,13^; in fact, bacteria, such as *Bacillus subtilis*, and endophytic fungi, such as *Phomopsis liquidambari*, have been used to control Fusarium wilt^14–16^. *Phanerochaetechrysosporium* is a typical representative of white rot fungi and belongs to the family Phanerochaetaceae in Polyporales, which is an order of fungi in the phylum Basidiomycota, subclass Agaricomycetidae^17^. The fungus is extensively used in environmental pollution control fields because it can degrade a wide variety of nonphenolic and phenolic compounds by producing ligninolytic enzymes^18^. Our previous work revealed the feasibility of overcoming continuous cropping problem through the use of *P*. *chrysosporium* ^19^. However, the impact of the fungus on the wilt disease of cut chrysanthemum is still not known. In the present study, *P*. *chrysosporium* was inoculated into a field that had been subjected to the long-term cropping of cut chrysanthemum. The effects of *P*. *chrysosporium* on wilt disease were then monitored. In addition, the microbial communities in the rhizosphere, phenolic acid degradation, and the interaction between *P*. *chrysosporium* and *F*. *oxysporum* were investigated to determine how *P*. *chrysosporium* affects the incidence of wilt disease. This study is helpful in understanding fungal function in reducing the wilt disease of cut chrysanthemum, especially because systematic studies on the control of the wilt disease of chrysanthemum by white rot fungus are rare.

## Results and Discussion

### Improvement of plant physiological status

**Figure 1:**
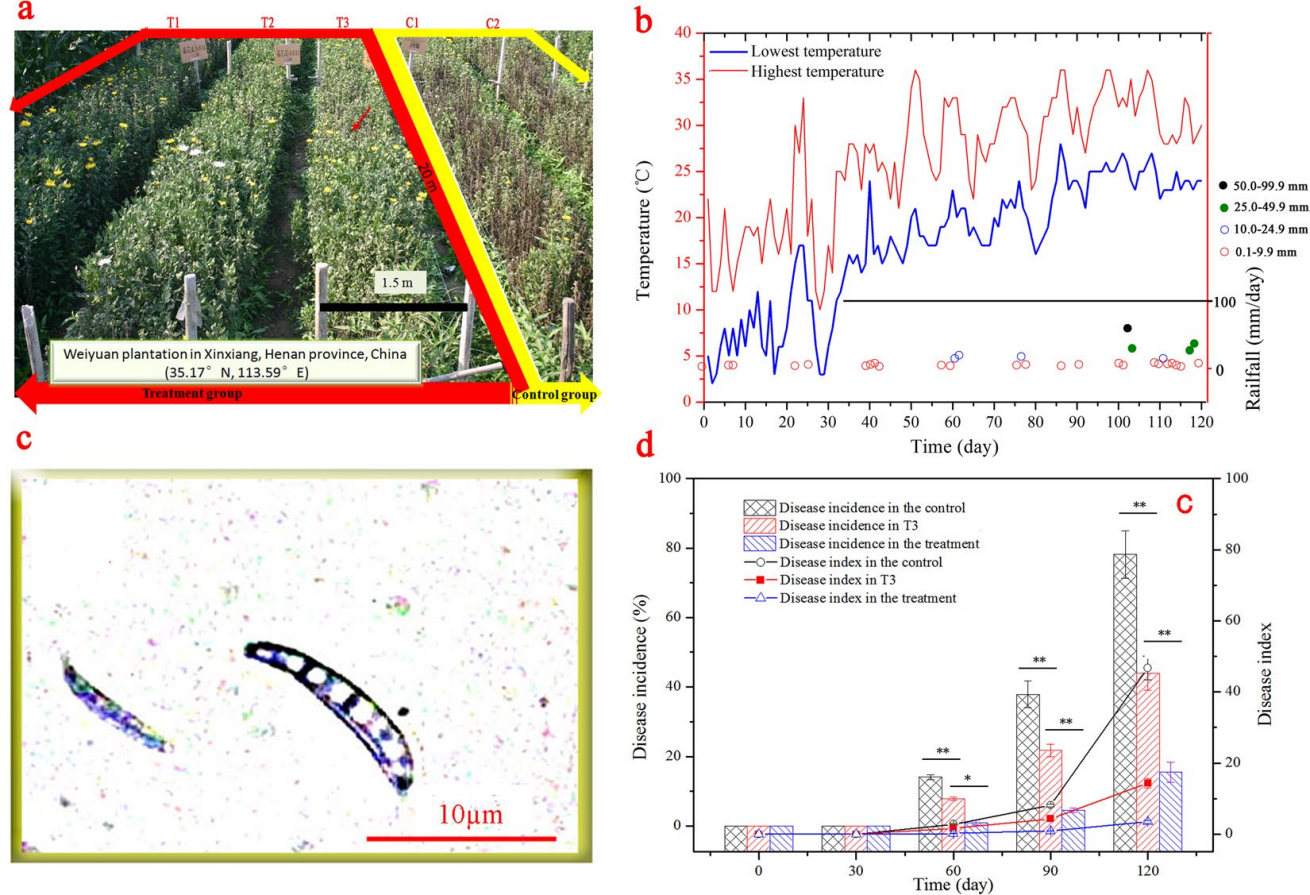
Comparisons of physiological status, wilt incidence, and disease index of cut chrysanthemum in the treatment and control. (**a)** Physiological status of cut chrysanthemum on day 120; (**b**) weather information; (**c**) micrograph of *F*. *oxysporum* under a microscope; (**d**) variations of wilt incidence and disease index of cut chrysanthemum during the cultivation. Mean values are given with error bars for ±SD (*n* = 4). * And ** correspondingly represent significance at the 0.05 and 0.01 levels in comparison with the control.

After the inoculation, the physiological status of cut chrysanthemum was significantly different in the control and treatment. At harvest time (on day 120; July 21, 2013), the plants in the control were all wilted (Fig. 1a). Most of them completely died, and massive losses in the flower yield were observed, as evidenced by the few flowers that matched market criteria. Compared with that in the control, the growth status of cut chrysanthemum in treated sections (T1 and T2) were dramatically better. On day 120, the average plant height, stem diameter, leaf length, leaf width, and chlorophyll content were all better than those of the control (Fig. S1), indicating that *P*. *chrysosporium* improved the plant growth in the five-year continuous cropping field.

### Control effect on wilt disease

*Fusarium oxysporum* caused severe disease during this experiment^20^, particularly in the summer because of the high temperature and rainfall that favor fungal growth and reproduction (Fig. 1b,c). After day 30, differences between wilt incidence and disease index in the treated and nontreated plots began to appear (Fig. 1d). On day 120, the incidence in the control was 78.16%, which was 5.03 times that in the treatment (15.45%). In addition, the disease index of the treatment, 3.45, was significantly lower than that of the control (46.70). Therefore, as a fungal biopesticide, *P*. *chrysosporium* significantly reduced the incidence and disease index of cut chrysanthemum. However, we should note that the cut chrysanthemum in section T3 was partly infected by *F*. *oxysporum* because this section was located next to the control, and the pathogen could be transmitted by soil. In addition, the *F*. *oxysporum* was isolated from the leaves and stems of infected plants (Fig. 1c), indicating the pathogen also can be transmitted by air. As shown in Fig. 1a, section T3 actually exhibited less disease in terms of both incidence (44.10%) and disease index (14.28%) on day 120 in comparison with the control. Thus, the treatment by *P*. *chrysosporium* clearly exhibited a control effect on the pathogen population present in the plot. Section T3 functioned as an isolation strip and played an important role in preventing the pathogen in the control from entering the treated aera (T1 and T2). In view of its specific function, the soil samples from T3 were not further analyzed in the subsequent tests.

**Figure 2:**
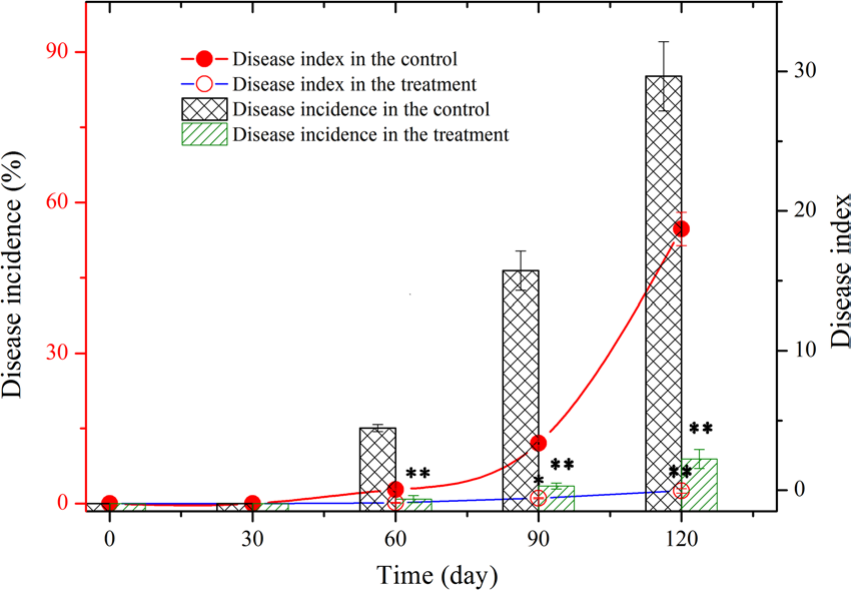
Variations of wilt incidence and disease index of cut chrysanthemum in the repeated experiment in 2014. Mean values are given with error bars for ±SD (n = 4). *p < 0.05; **p < 0.01.

Another experiment was also carried out in 2014, and the results revealed the repeatability of the control of Fusarium wilt by *P*. *chrysosporium*. Compared with those in the first season, the incidence and disease index in the treatment were relatively lower (Fig. 2). On day 120, the disease incidence and index in the treatment were 8.9 % and 2.55, respectively, suggesting that the continuous use of *P*. *chrysosporium* is helpful to improve the control of wilt disease.

### Effect on the bacterial community structure in soil

A total of 227,382 trimmed sequences with an average sequence length of 396 bp were obtained. The operational taxonomic units (OTUs) identified for 11 samples are shown in Table 1. Good’s coverage indicated that 94.42–98.46% of the species in the samples were recovered at a cutoff of 97% sequence similarity. However, the slope of the taxon rarefaction curves remained incomplete, thereby suggesting that the true bacterial diversity likely exceeded the current perceptions (Fig. S2a). Chao1 was used to estimate bacterial richness^21^, and the initial Chao1 index of soil before cultivation was 1950. On day 15, the Chao1value of the treatment decreased to 1817, which was lower than that of the control (2194). This phenomenon is attributed to the presence of *P*. *chrysosporium* in soil. After day 15, the Chao1 index of the treatment recovered to 2211, and during the left cultivation, the value surpassed that of the control, except on day 90. Therefore, *P*. *chrysosporium* finally increased bacterial richness. The Shannon index, which represents bacterial diversity^22^, indicated the same change trend as that of the Chao1 estimator. Before day 30, the treatment had a lower Shannon index than the control, indicating that *P*. *chrysosporium* first reduced the bacterial community diversity. However, bacterial diversity in the treatment recovered gradually. On day 120, the indexes of the two groups were 6.64 and 6.60, indicating no significant difference in bacterial community diversity.

**Table 1:** Sequencing results and statistical analysis of soil samples.

Universal primers	Samples	Reads	OTUs	Coverage (%)	Chao 1 estimator	Shannon diversity index
515F-907R	C0	24471	1662	98.46	1950	6.12
	C15	17692	1904	97.55	2194	6.54
	T15	10753	1278	95.11	1817	4.84
	C30	9952	1472	94.42	2048	5.98
	T30	17128	1688	96.91	2211	5.92
	C60	19737	1675	97.64	2039	5.90
	T60	17909	1689	97.26	2083	6.24
	C90	18960	1989	97.36	2384	6.56
	T90	15339	1802	96.99	2147	6.43
	C120	22041	1969	98.25	2232	6.60
	T120	16750	1915	97.16	2288	6.64
1737F-2043R	C0	28162	278	99.91	292	3.56
	C15	19475	461	99.70	501	4.22
	T15	21291	460	99.78	492	4.71
	C30	22587	407	99.73	453	4.13
	T30	22402	397	99.66	460	3.41
	C60	18769	301	99.62	351	2.49
	T60	25405	542	99.57	608	3.78
	C90	15266	414	99.67	452	4.36
	T90	19287	388	99.79	417	4.37
	C120	9521	233	99.56	270	3.73
	T120	14678	543	99.47	584	4.76

C0: The intial soil sample before cultivation

C15–C120: Soil samples obtained in the control group in days 15–120;

T15–T120: Soil samples obtained in the treatment group in days 15–120.

**Figure 3:**
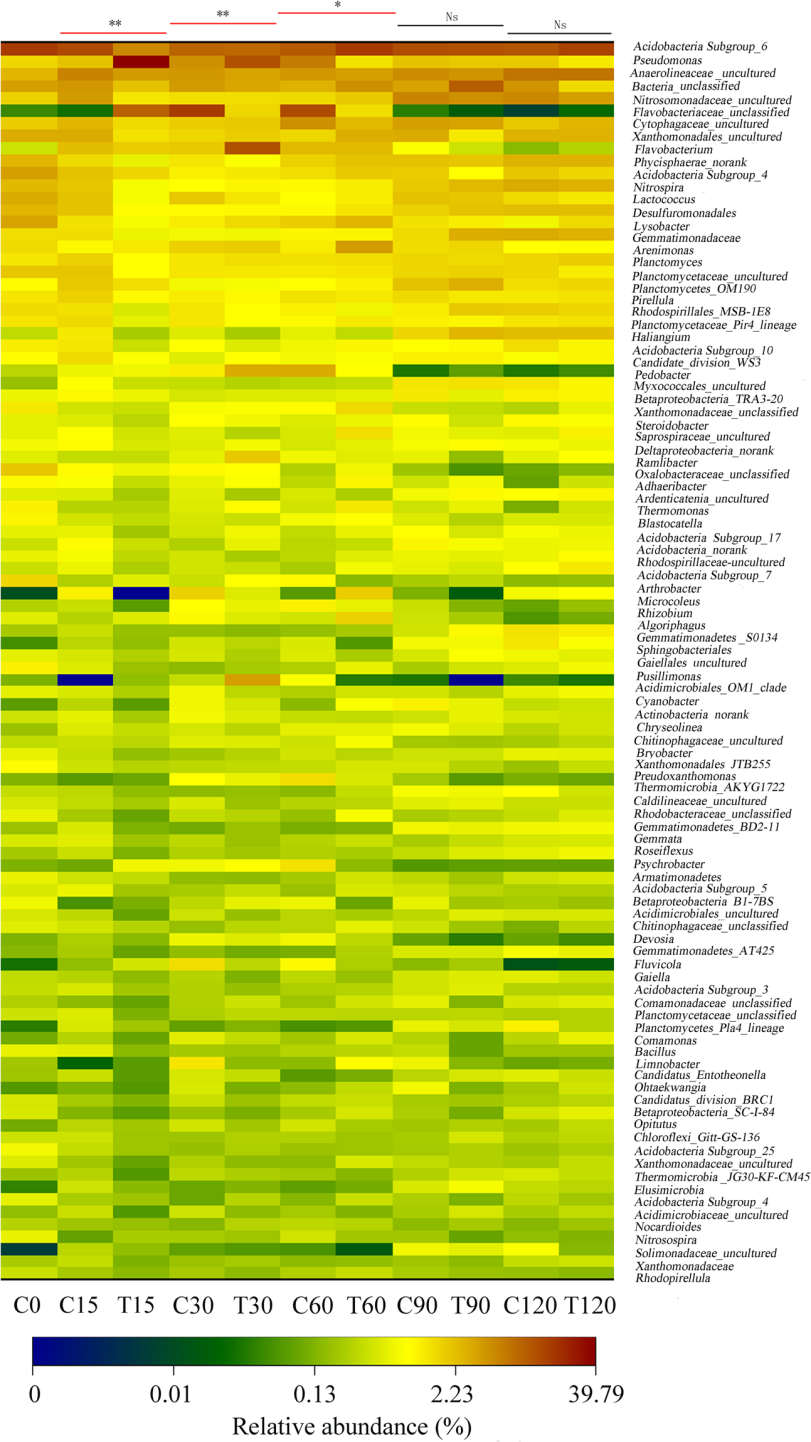
Heat map illustrating the RA change of the top 100 genera in soil samples. The color scale indicates the magnitude of RA. Ns, non-significant; *p < 0.05; **p < 0.01.

**Figure 4:**
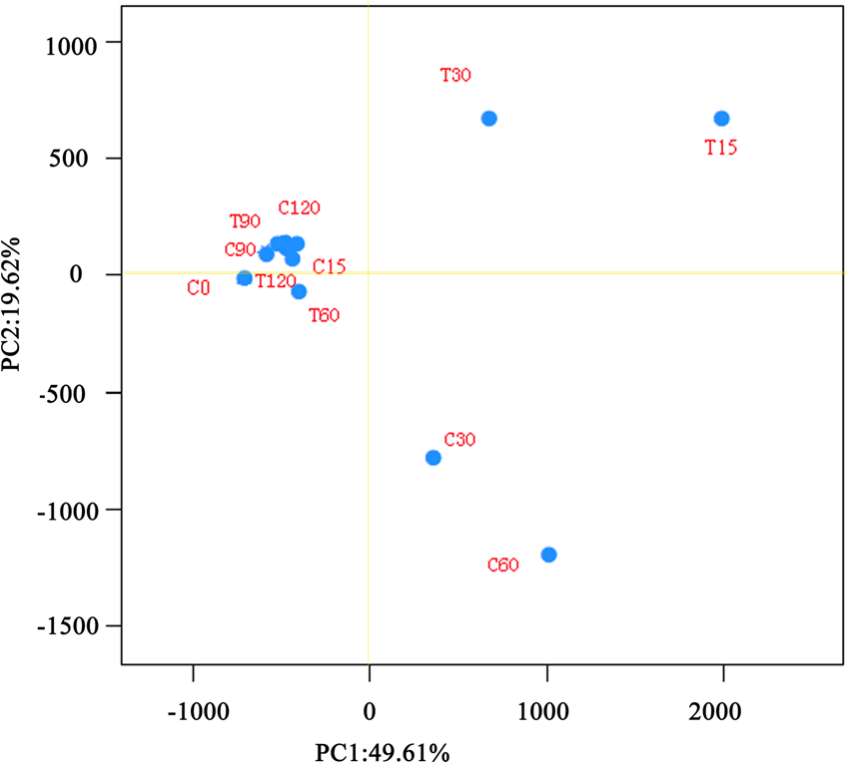
Principal component analysis of the variance of bacterial communities in different soil samples. The figure describes the samples C15-C60 and T15-T60 as clearly different from each other.

The bacterial OTUs of the soil samples were grouped into 19 phyla, and more than 97.9% of the total relative abundance (RA) of *Actinobacteria*, *Bacteroidetes*, *Planctomycetes*, and *Proteobacteria* suggested that they were the main bacteria in soil (Fig. S3). At the phylum level, the significant difference in RA appeared on day 15 (*p* < 0.01). After day 60, the difference in bacterial community structure between the control and the treatment tended to be non-significant, and on days 90 and 120, no statistically significant difference was observed at the 0.05 level. At the genus level, the OTUs in the soil samples were divided into 536 genera, and the top 100 genera that occupied 96.33–99.29% of the total RA presented an ecological succession of the bacterial communities during the 120-day cultivation (Fig. 3). Dissimilarity analyses of the soil samples from the two groups also showed that *P*. *chrysosporium* only changed the bacterial community structure at the first half stage (before day 60) and that the differences in community structures were not significant (*p*  >  0.05 on days 90 and 120). The conclusion was also supported by the results of the PCA analysis. The PCA score plot (Fig. 4) revealed that the structures of soil bacterial communities between the control (C15-C60) and the treatment (T15-T60) were clearly different. Nevertheless, after day 60, their structures in two groups (C90 and T90; C129 and T120) were almost similar to those in the initial soil before cultivation (C0).

### Effect on eukaryotic community structure in soil

Eukaryotic sequences with an average sequence length of 334 bp were identified as 1561 OTUs via phylogenic analysis. Good’s coverages (99.47–99.91%) at 97% similarity indicated that the sequencing results were sufficient to represent the natural eukaryotic community (Table 1). The rarefaction curve also revealed that the overall eukaryotic diversity was well exhibited (Fig. S2b). The eukaryotic Chao1 estimators in both groups increased on day 15, and during the cultivation, the estimator of the treatment was higher than that of the control, except on day 90. On day 120, the richness of the treatment considerably increased, and the Chao1 estimator was 2.16 times that of the control. Nonetheless, the eukaryotic community diversity in soil was changed by *P*. *chrysosporium*, and the treatment had a higher Shannon diversity index than the control, except on day 30. The changes in community richness and diversity indicated that *P*. *chrysosporium* finally promoted eukaryotic propagation.

**Figure 5:**
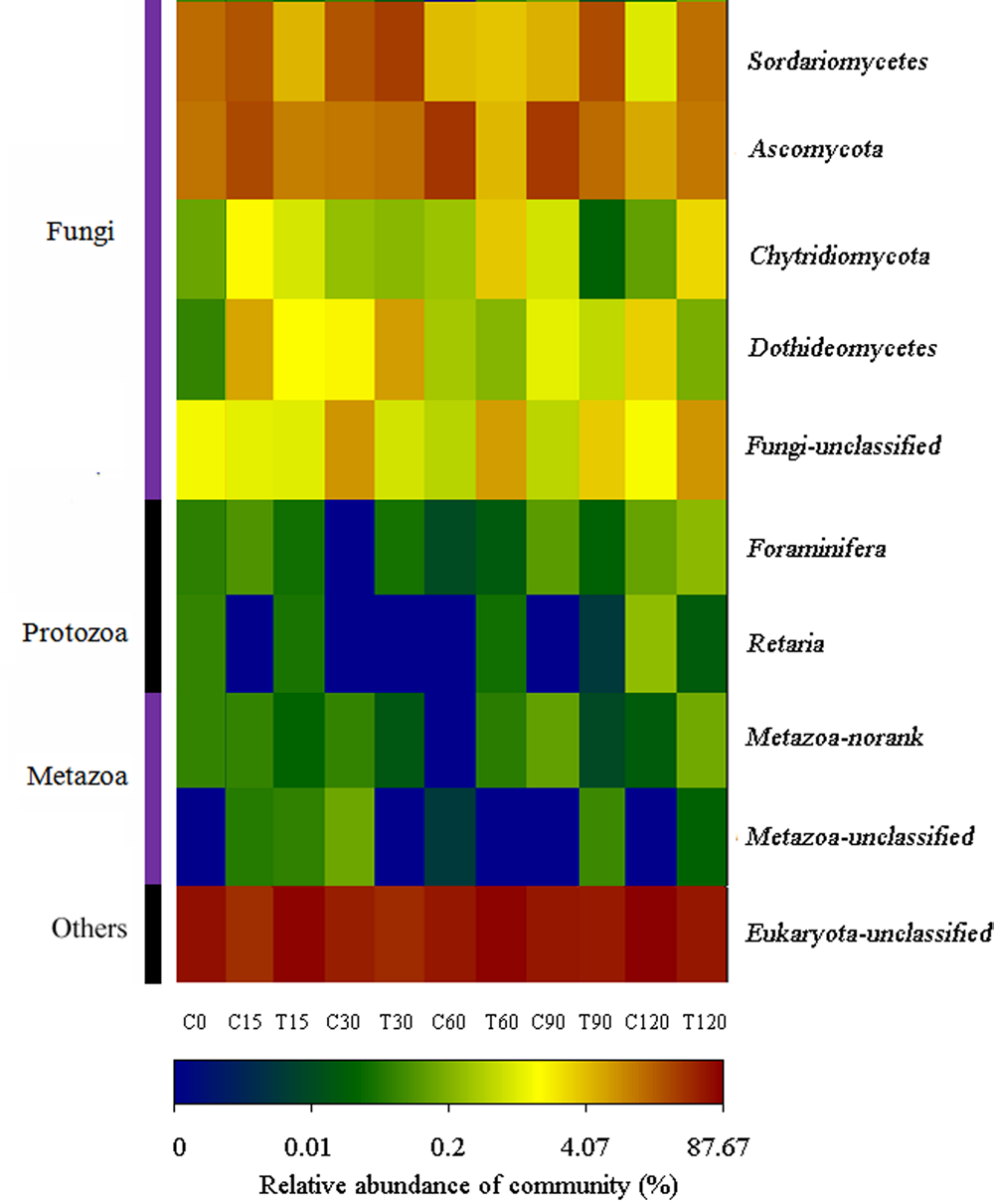
Heat map illustrating the RA change of the eukaryotes in the soil samples. The color scale indicates the magnitude of RA.

Eukaryotes consist of fungi, protozoa, metazoan, and so on. In this work, the eukaryotic community structure was investigated at the class level. OTUs were grouped into 10 classes (Fig. 5) containing fungi (5 classes), protozoa (2 classes), metazoa (2 classes), and unclassified eukaryotes; fungi were the main eukaryotes in the soil samples. Dissimilarity analysis showed the differences in the fungal community structures during the whole cultivation (*p* < 0.01). However, due to the limited accuracy of molecular identification based on 18S rDNA analysis^23^, many eukaryotic OTUs were not properly classified even at the class level. Information from metagenomic analysis was not enough to accurately reflect the profile of the fungal community. Thus, the change in fungal quantity, especially that of *F*. *oxysporum*, needs to be further investigated with a different method.

### Effect on bacterial and fungal quantity

**Figure 6:**
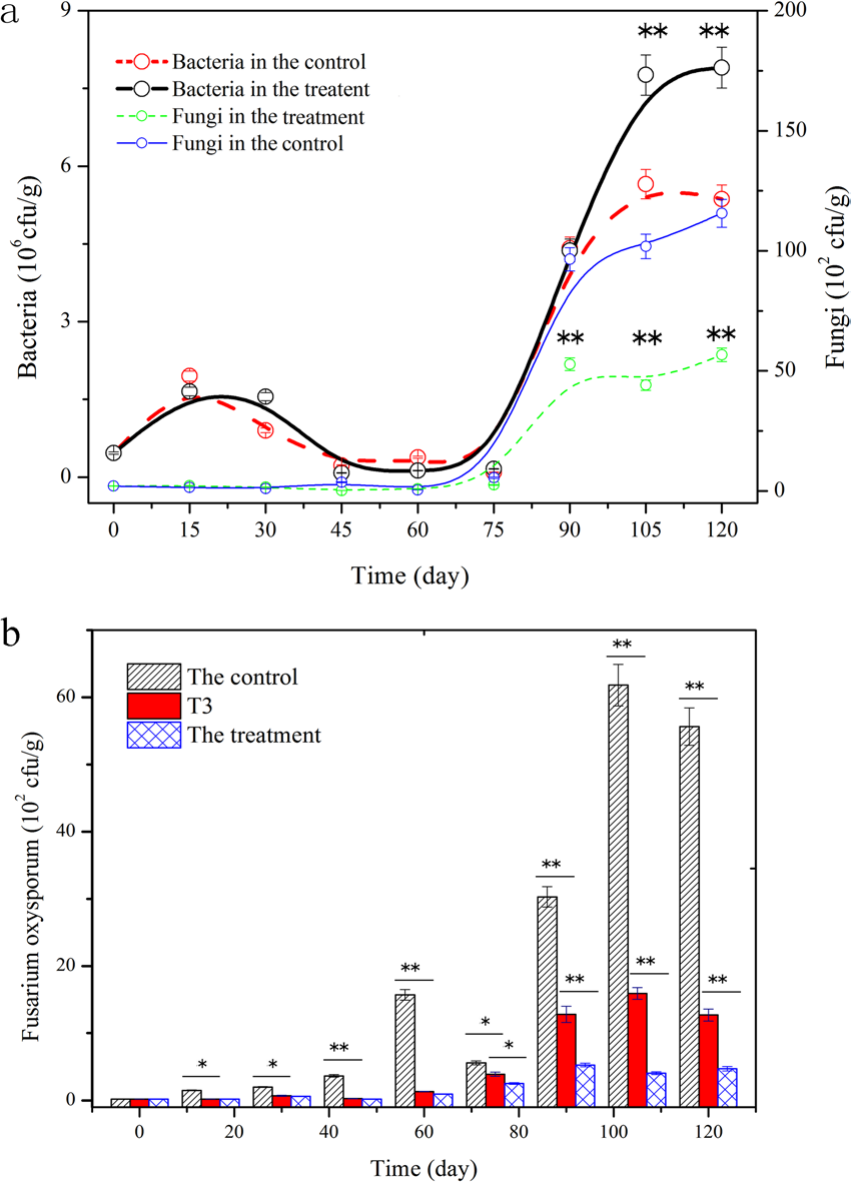
Enumeration results of bacteria, fungi, and *F*. *oxysporum* in soil during cultivation. (**a**) Variation of bacterial and fungal quantity with time; (**b**) variation of the number of *F*. *oxysporum* with time. Mean values are given with error bars for ±SD (*n* = 5). *p < 0.05; **p < 0.01.

Figure 6a shows that the bacterial quantities in both groups exhibited a similar fluctuation (increased first and then declined) before day 60 and that the differences in quantity were not significant. After day 60, the bacterial quantities started to increase sharply. In the treatment, it reached 7.9 × 10^6^ cfu/g on day 120; this value exceeded that in the control (5.4 × 10^6^ cfu/g), thus indicating that *P*. *chrysosporium* increased the bacterial quantity in soil. This conclusion is in accordance with the previous Chao1 analysis. The fungal quantity showed no clear change before day 60, and after day 60, it started to increase. On day 120, the fungal quantity in the control was 2.04 times that in the treatment^24^.

The wilt disease of cut chrysanthemum can be spread by soil, and *F*. *oxysporum* intrudes from the root, resulting in plant death^25,26^. The absence of severe disease in the treatment (T1 and T2) was attributed to the following: (i) the appearance of *P*. *chrysosporium* improved the immune ability of cut chrysanthemum such that *F*. *oxysporum* could not intrude; however, the speculation is not acceptable because the wilt disease occurred in T3, which was also inoculated with *P*. *chrysosporium*; and (ii) in the treatment (T1 and T2), the appearance of *P*. *chrysosporium* in soil reduced or eliminated the pathogens of wilt disease. To validate this point, we counted the quantity of *F*. *oxysporum* in soil during the cultivation. The quantity of *F*. *oxysporum* in the treatment was significantly low (*p* < 0.01), and after day 90, it was approximately one-tenth of that in the control (Fig. 6b). Moreover, the population of *F*. *oxysporum* before day 60 showed that the quantity of *F*. *oxysporum* in T3 was not different from that in T1 and T2 (*p* > 0.05), although it was less than that of the control. This result indicated that the treatment by *P*. *chrysosporium* effectively inhibited the growth of *F*. *oxysporum* in soil. After day 60, the increase of *F*. *oxysporum* population in T3 was attributed to the pathogens transmitted by air and soil. Obviously, the reduction of *F*. *oxysporum* in the soil as a result of the addition of *P*. *chrysosporium* served as the key factor explaining the low wilt incidence in the treatment.

### Inhibition against F. oxysporum due to P. chrysosporium

**Figure 7:**
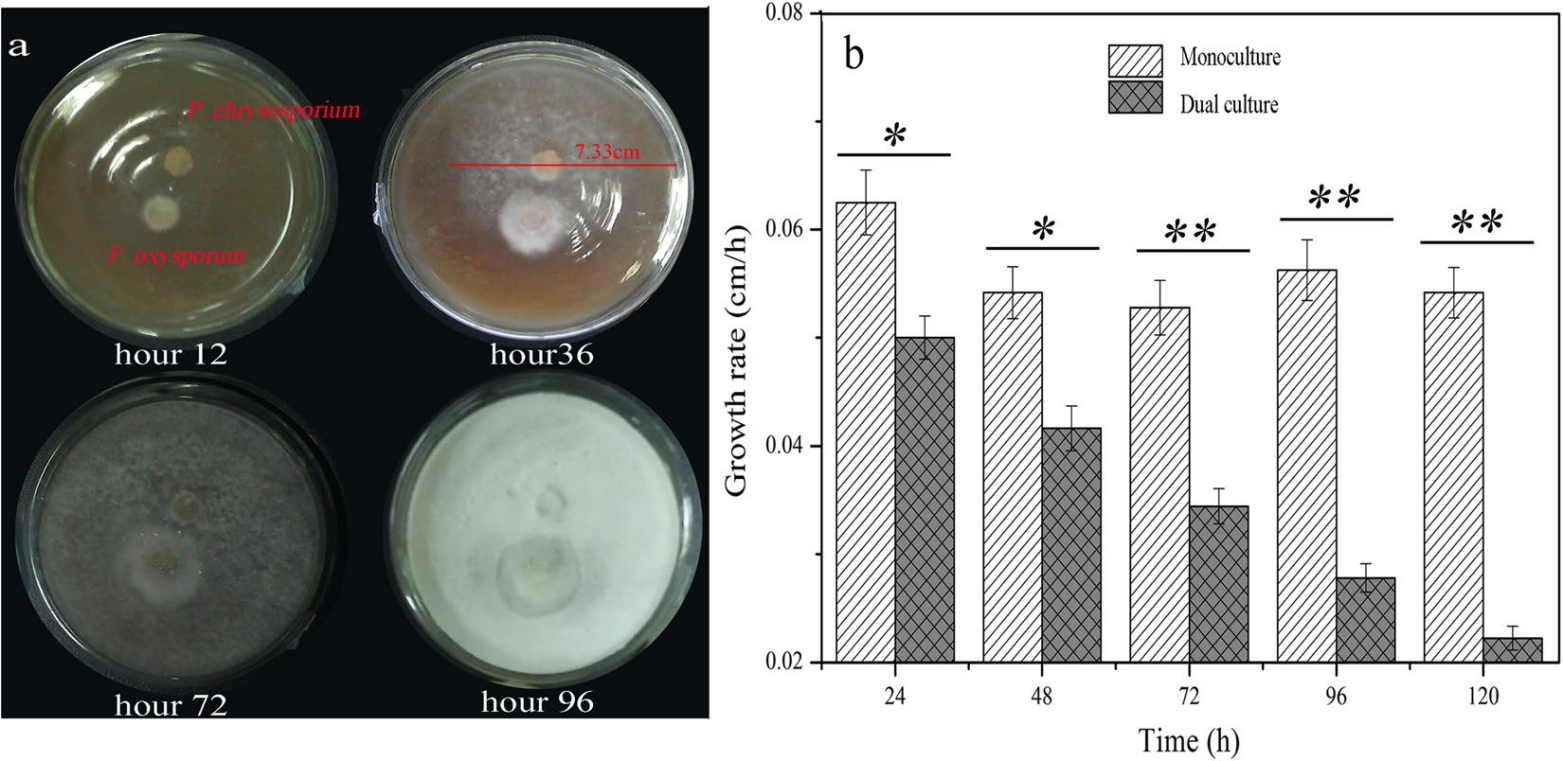
Dual culture of *F*. *oxysporum* and *P*. *chrysosporium* and growth rates of *F*. *oxysporum*. (**a**) Dual culture of *F*. *oxysporum* and *P*. *chrysosporium* on solid plate; (**b**) growth rates of *F*. *oxysporum* during the monoculture and dual culture. Mean values ±SD are given (*n* = 4). *p < 0.05; **p < 0.01.

The analysis of *F*. *oxysporum* showed that the pathogen quantity in the soil was reduced sharply. However, the exact mechanism of how *P*. *chrysosporium* reduced the pathogen quantity is still unclear. Several types of ecological relationships exist among microbes, and they include symbiosis, syntrophism, and inhibition^27,28^. In the dual culture of *P*. *chrysosporium* and *F*. *oxysporum*, the clear inhibition against *F*. *oxysporum* caused by *P*. *chrysosporium* was observed (Fig. 7a). However, the secretion of *P*. *chrysosporium* exerted no inhibition effect on *F*. *oxysporum*, showing that *P*. *chrysosporium* did not inhibit the growth of *F*. *oxysporum* by means of antibiotic production. *P*. *chrysosporium* exhibited a higher growth rate than *F*. *oxysporum*. At hour 48, its colony diameter reached 7.33 cm, and the growth rate of 0.20 cm/h was 2.8 times that of *F*. *oxysporum*. In addition, the growth rates of *F*. *oxysporum* in the monoculture and dual culture indicated that the fungus was inhibited by *P*. *chrysosporium* (Fig. 7b). Thus, we deduced that *P*. *chrysosporium* propagated quickly and restricted the growth of *F*. *oxysporum* by competing for resources, including nutrients and space, because the relationship is natural and universal between co-cultured microbes^29^. Other mechanisms, such as hyphae interaction, are also probable^30^, although the details need to be further studied.

**Figure 8:**
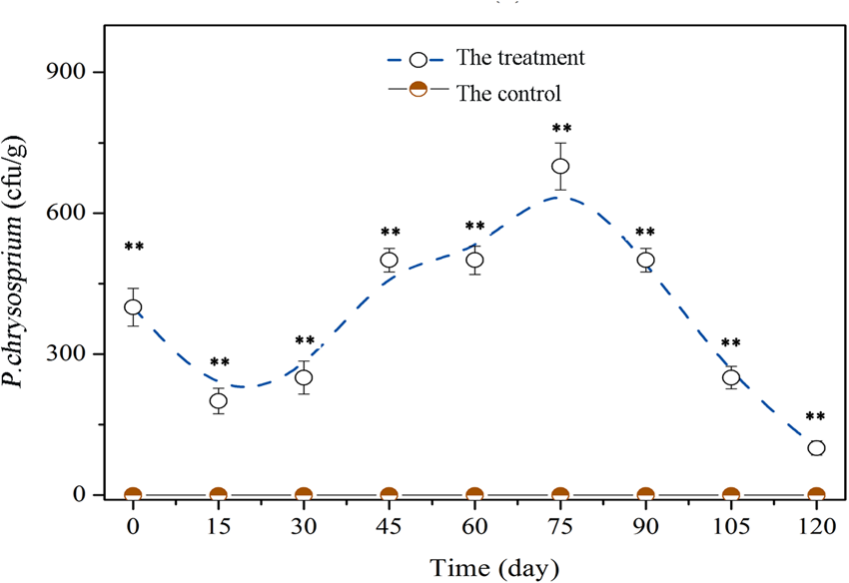
Quantity of *P*. *chrysosporium* in soil varying with time during the 120-day cultivation. Mean values ±SD are given (*n* = 4). * and ** correspondingly represent significance at the 0.05 and 0.01 levels in comparison with the control.

*P*. *chrysosporium* is not an indigenous fungus in soil. In this work, its quantity was counted by colony enumeration because a molecular method cannot accurately identify the fungus at the species level. The fungal quantity in the treatment decreased on day 15 and then increased and reached the peak value on day 75 (Fig. 8). Thus, *P*. *chrysosporium* persisted, although it offered few advantages over the majority of bacteria with a high reproduction rate in the ecological competition in the soil. As a result of the inhibition caused by *P*. *chrysosporium*, the quantity of *F*. *oxysporum* in the treatment was significantly lower than that in the control during the whole cultivation (Fig. 6b). This result can be used to explain why no severe wilt disease occurred in the treatment.

### Effects of degradation of phenolic acids on wilt disease

**Figure 9:**
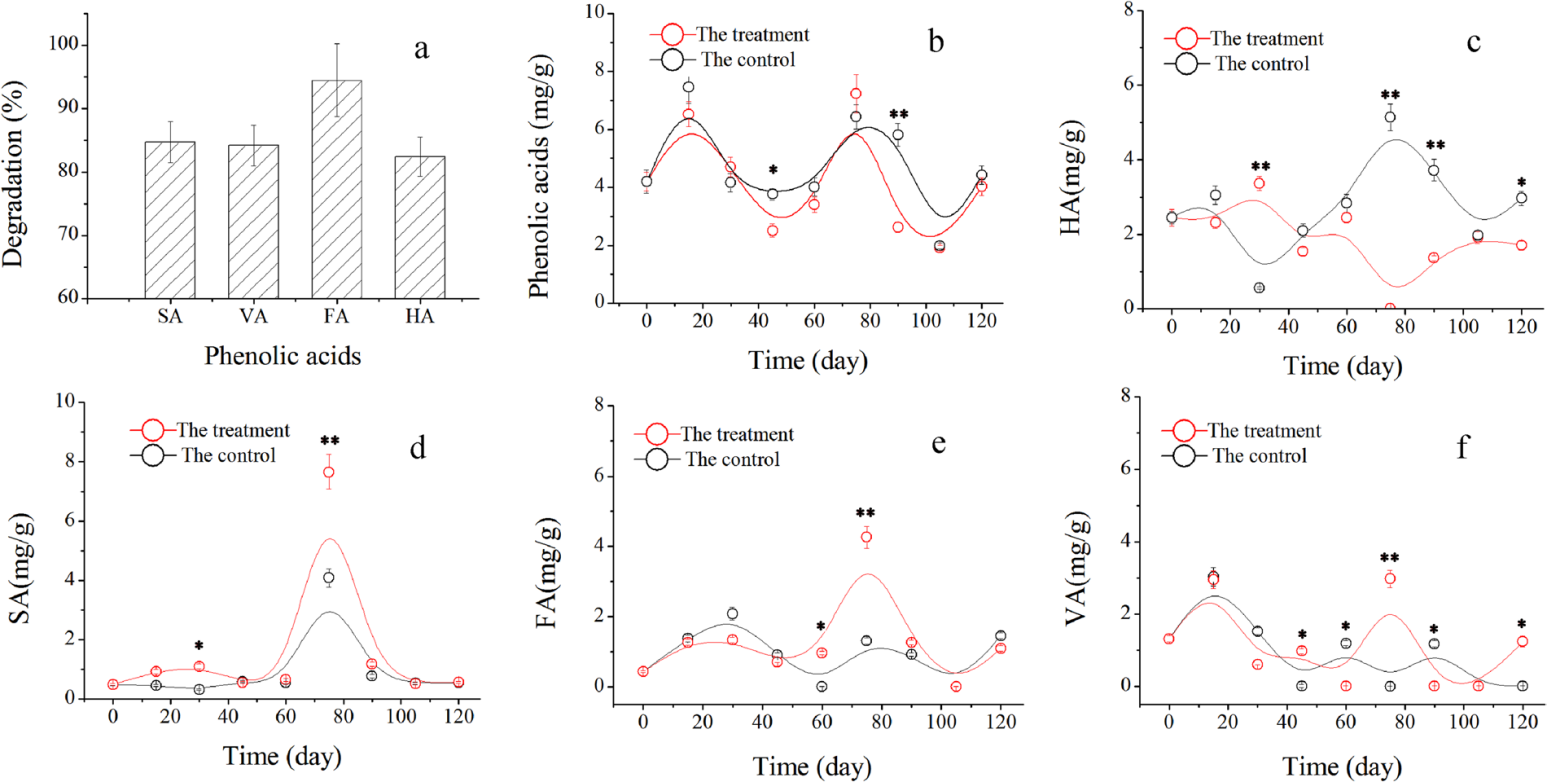
Degradation of phenolic acids by *P*. *chrysosporium*. (**a**) Degradation rates of syringic acid (SA), vanillic acid (VA), ferulic acid (FA), and p-hydroxybenzoic acid (HA) in submerged cultivation; (**b**) degradation rate of total phenolic acids in soil; (**c**) variation of HA with time; (**d**) variation of SA with time; (**e**) variation of FA with time; (**f**) variation of VA with time in the control and treatment. Mean values ±SD are given (*n* = 4). * and ** correspondingly represent significance at the 0.05 and 0.01 levels in comparison with the control.

Generally, autotoxicity explains why continuously cropped plants are easy to be infected by *F*. *oxysporum*, and several toxic phenolic acids that plants secret are reported to be highly toxic to plants or helpful for the reproduction of *F*. *oxysporum* ^20,31^. According to a seven-year investigation we conducted, syringic acid (SA), vanillic acid (VA), ferulic acid (FA), and p-hydroxybenzoic acid (HA) are the main phenolic acids in soils under different durations of continuous planting of cut chrysanthemum (Fig. S4). *P*. *chrysosporium* can degrade phenolic acids through its ligninolytic enzymes^32,33^, and after 72 h of degradation in submerged culture, the degradation rates of SA, VA, FA, and HA reach 84.7%, 84.2%, 94.5%, and 82.4%, respectively, suggesting the high ability of *P*. *chrysosporium* to degrade phenolic acids (Fig. 9a).

According to the degradation data, when the fungus was sprayed into the soil, the total phenolic acids in the treatment decreased in comparison with that in the control, and *P*. *chrysosporium* reduced the phenolic acids in soil, especially on days 45 and 90 (Fig. 9b). However, the analysis of the degradation of phenolic acids in the two groups showed that the addition of *P*. *chrysosporium* only significantly improved the degradation of HA (*p* < 0.05) and failed to benefit the degradation of SA, VA, and FA (Fig. 9c–f). The probable reasons are as follows: (i) the contents of phenolic acids in the process of plant growth are variable, and plants secrete various phenolic acids at different growth phases^34^. For example, the sharp enhancement of FA, SA, and VA on day 75 indicated that they were abundantly produced by the plants at this phase. In addition, microorganisms, such as bacteria in soil, produce these phenolic acids through their metabolic systems^35^; (ii) although the soil was inoculated with *P*. *chrysosporium*, the degradation of phenolic acids could not be entirely attributed to the fungus because of the large number of indigenous microorganisms in the soil, which are also able to remove various phenolic acids. Given the change in the soil microbial community structure, the discrepant degradations of phenolic acids in soil are understandable.

**Figure 10:**
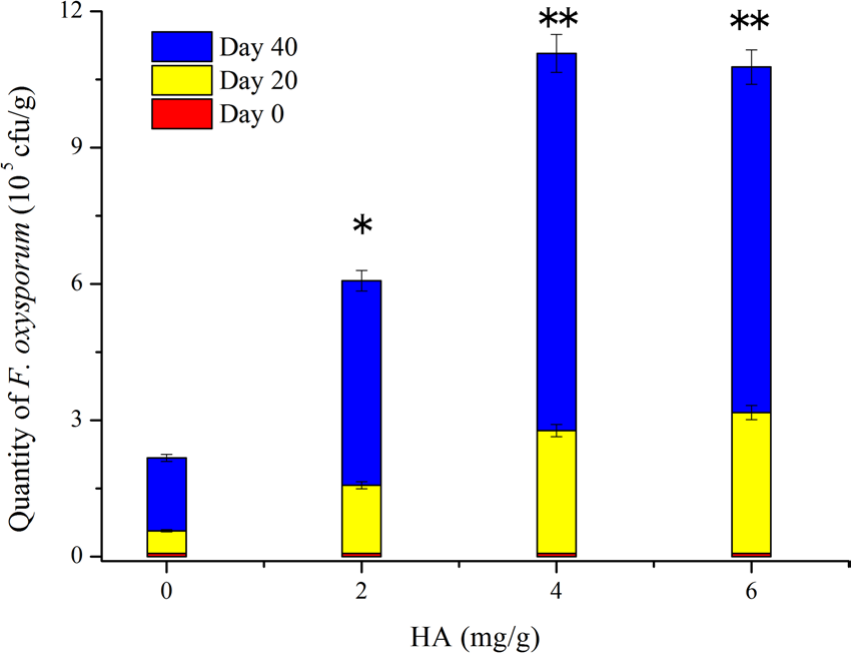
Effect of HA of different concentrations on *F*. *oxysporum* quantity in pot experiment. Mean values are given with error bars for ±SD (*n* = 10). *p < 0.05; **p < 0.01.

Interestingly, the pot experiment confirmed that 2.0–6.0 mg/g of HA promoted the growth of *F*. *oxysporum* (Fig. 10). In comparison with those in the pots without HA, the quantities of *F*. *oxysporum* in the pots supplied with 4 and 6 mg/g of HA were increased significantly (*p* < 0.01). Therefore, the decrease of HA content can also be used to explain the alleviation of wilt disease in the treatment.

### Effects of other factors on wilt disease

**Figure 11:**
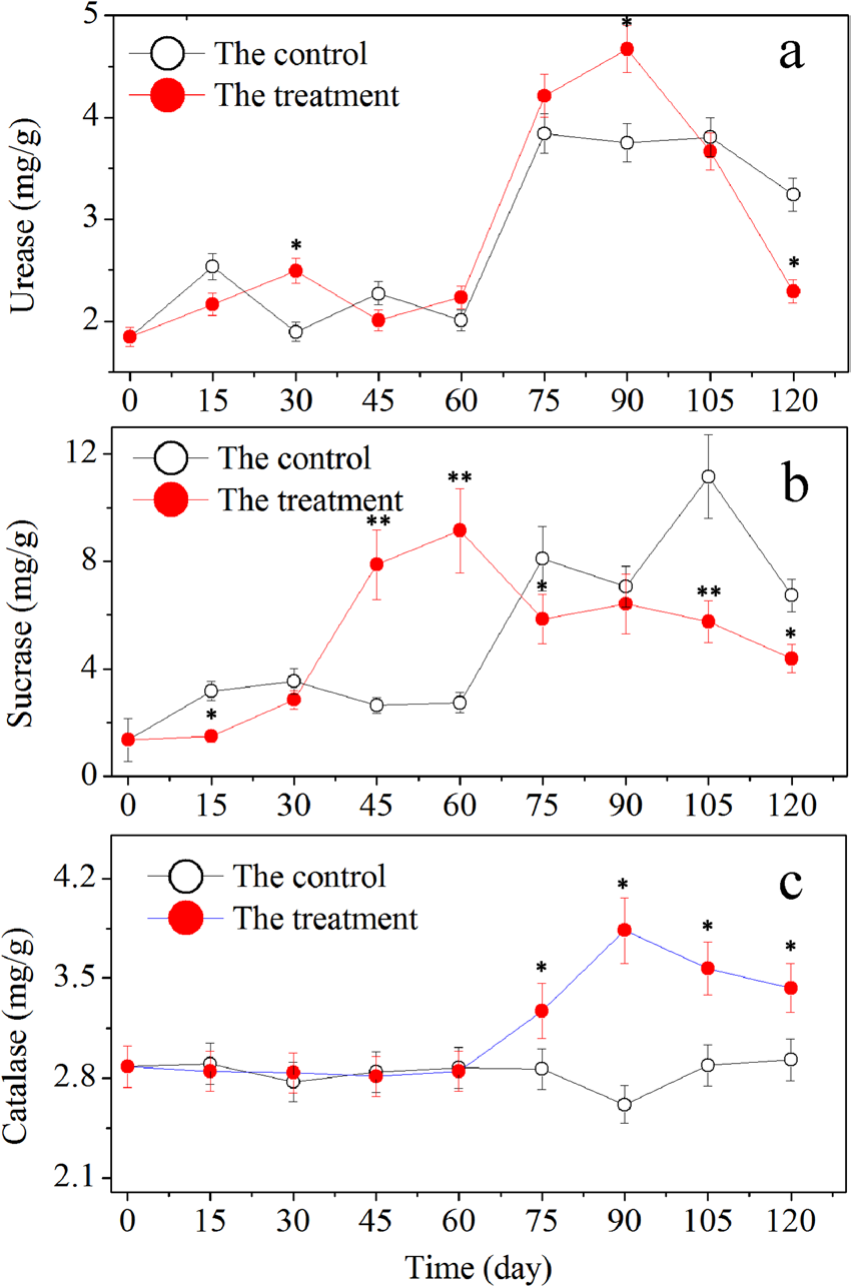
Changes of urease, sucrase, and catalase activities in the rhizosphere. Mean values are given with error bars for ±SD. * and ** correspondingly represent significance at the 0.05 and 0.01 levels in comparison with the control.

Soil is an extremely complicated ecosystem. Thus, the inhibition caused by *P*. *chrysosporium* and the change of HA in soil are not the sole reasons that could explain the wilt incidence observed in this study. Other factors, including nutrients and the physical and chemical properties of soil, could also influence wilt incidence. For example, nutrients may result in the change of wilt incidence by affecting the immunity of plants. In this experiment, the changes in the quantity and structure of the microbial community undoubtedly revealed the different activities of enzymes, including urease, sucrase, and catalase in the rhizosphere (Fig. 11). The latter led to the nutrient changes and the toxic effect of hydrogen peroxide in soil^19^, and it affected both plant and pathogen growth. Certainly, additional evidence is needed to prove the effects of these factors on wilt disease of cut chrysanthemum. The resulting information will surely broaden our understanding of the mechanism of alleviation of Fusarium wilt caused by *P*. *chrysosporium*.

## Materials and Methods

### Chemicals and microorganism

All of the chemicals used in this study were of analytical grade unless otherwise stated. The standard compounds of *p*-hydroxybenzoic acid, vanillic acid, syringic acid, and ferulic acid were obtained from Sigma Company. Both *P*. *chrysosporium* (ATCC24725) and *F*. *oxysporum* (ATCC48112^TM^) were obtained from the Henan Province Engineering Laboratory for Bioconversion Technology of Functional Microbes, China.

### Cultivation of P. chrysosporium and F. oxysporum mycelia

Both *P*. *chrysosporium* and *F*. *oxysporum* were incubated on potato dextrose agar (PDA) plates and sub-cultured for three days at 30 °C. Subsequently, conidium suspensions (5 × 10^7^ spores/mL) were prepared in sterile water. The suspensions (5.0 mL/flask) were inoculated to 500-mL flasks containing liquid medium (200 mL) composed of the following: 10.0 g/L of glucose, 2.5 g/L of (NH_4_)_2_SO_4_, 1.0 g/L of KH_2_PO_4_, 0.8 g/L of MgSO_4_, 0.5 g/L of CaCl_2_, and 0.7% trace element solution containing 0.5 g/L of glycine, 0.1 g/L of FeSO_4_·7H_2_O, 0.1 g/L of CoSO_4_, 0.1 g/L of ZnSO_4_, 10 mg/L of CuSO_4_·5H_2_O, 10 mg/L of AlK(SO_4_)_2_·12H_2_O, 10 mg/L of H_2_BO_3_, and 10 mg/L of Na_2_MoO_2_·2H_2_O. The flasks were placed in a shaking incubator at 200 rpm and 30 °C. After a four-day submerged cultivation, the mycelial pellets in fermentation broth (5.6 g/L for *P*. *chrysosporium* and 4.9 g/L for *F*. *oxysporum*; dry weight) were smashed in a homogenizer for 5 min at 10000 rpm and 25 °C. The obtained hyphal suspensions of *P*. *chrysosporium* and *F*. *oxysporum* were diluted with sterile water and used as inocula in the corresponding experiments.

### Experimental site and description

The experimental site was located in Weiyuan plantation in Xinxiang, Henan province, China (35.17°N, 113.59°E), where the quality of cut chrysanthemum was seriously affected by wilt disease caused by *F*. *oxysporum*. After five years of continuous cropping, the yield was lower than 10% of that in the first year. The planting plot used for this experiment consisted of five sections, including the treatments (T1, T2, and T3) and controls (C1 and C2) with 0.5 m between each section. In the treated plots, 600 g/acre of shattered mycelia of *P*. *chrysosporium* was sprayed on the surface of the soil three days before chrysanthemum planting. In the control sections, no mycelium was sprayed. T3, located between two treated sections (T1 and T2) and control (C1 and C2), was defined as the isolation strip. The experiments were conducted from March to July in 2013 and then repeated in 2014. The plot is shown in Fig. 1a, a photograph taken 120 days after planting. The detailed weather information including temperature and rainfall is shown in Fig. 1b.

### 16S rDNA sequencing and bioinformatic analysis

Soil samples collected from the treatment (T1 and T2) and control (C1 and C2) sections were taken from 5–10 cm below the surface and 5 cm away from the plants. At least five samples were taken from each plot at each time, and biochemical indices, such as phenolic acids, were documented^36,37^. Metagenomic DNA of soil samples was extracted with the MoBio DNA Power Soil Kit. DNA purity and concentration were evaluated on a spectrophotometer (Nano Drop Technologies, USA) at 260, 280, and 230 nm, and they were visualized on an agarose gel (1.0%) containing ethidium bromide. PCR amplifications of the highly variable V1–V3 regions of the bacterial 16S rRNA gene and V4 region of the eukaryotic 18S rRNA gene were conducted based on the universal primer pairs (515F and 907R) and (1737F and 2043R), respectively. The thermo-cycling procedure consisted of an initial denaturation step at 95 °C for 3 min, followed by 25 cycles (16S rRNA) or 30 cycles (18S rRNA), where each cycle was set to 95 °C for 30 s (denaturation), 55 °C for 30 s (annealing), 72 °C for 30 s (extension), and a final extension at 72 °C for 5 min. Each reaction was conducted in 20 µL of reaction mixtures containing 10 ng of template DNA, 5 μM of each of the primers, 2.5 mM of the deoxynucleoside triphosphate mix, and 1 unit of Transstart Fastpfu Polymerase (TransGen Biotech, China). The PCR cycling reactions were performed in a GeneAmp®9700DNA thermocycler (ABI, USA), and the amplified products were visualized on agarose gels (2.0% in tris–borate–ethylenediaminetetraacetic acid buffer) containing EB and purified with a DNA gel extraction kit (Axygen Inc., USA). The DNA concentration of each PCR product was determined prior to sequencing, and the amplicons from each PCR reaction were pooled together in equimolar ratios to reduce the biases of each individual reaction and then subjected to emulsion PCR to generate amplicon libraries. Deep sequencing was conducted on a Roche Genome GS FLX system at Shanghai Majorbio Pharmaceutical Technology Co., Ltd, China. The metagenomic data were deposited in the National Center for Biotechnology Information (NCBI) Sequence Read Archive under accession number SRP102329.

The results obtained from the sequencer were output in the form of standard flowgram format files. Raw sequencing data were quality-filtered to remove the short or low-quality reads. Any sequence with more than two base mismatches determined by checking with the forward primer or with a similarity of less than 95% between the reverse primer and adaptor was discarded via Seqcln software analysis. The low-quality sequences containing ambiguous bases were further trimmed with the Mothur software. The “dist.seqs” command was performed to identify OTUs by 97% sequence similarity. The trimmed sequences were subjected to Megablast and searched against SILVA aligned 16S/18S small subunit rRNA sequence database to acquire a high taxonomic resolution. Analyses on rarefaction, Shannon diversity index, and Chao1 richness were performed with the Mothur software^38^.

### Bacterial and fungal quantity

Each soil sample (10 g) was mixed with 90 mL of sterile water and then diluted to different concentration gradients. Both bacterial and fungal quantity were determined with the plate cultivation method^39^, and 100 μL of diluents were incubated on suitable media (bacteria were cultivated on beef extract–peptone agar medium for one day at 37 °C, and fungi were cultivated on Martin medium for two to three days at 28 °C). Three replications were performed on each of the soil samples.

### Dual culture test

Both *P*. *chrysosporium* and *F*. *oxysporum* were cultivated on a solid medium (SD) plate at 30 °C. SD consisted of 10 g/L of glucose, 200 g/L of potato, 20 g/L of agar powder, and 100 mL of leach liquor of soil per liter. Leach liquor of soil was prepared with 10 g of soil from the field subjected to five years of continuous cropping and 200 mL of deionized water. The soil was soaked for 48 h and then filtered, and the filter liquor was concentrated to 100 mL. The diameters of fungal colonies were measured, and the growth rates of *P*. *chrysosporium* and *F*. *oxysporum* were calculated on the basis of colony diameter. *P*. *chrysosporium* was also monocultivated in a flask (500 mL) containing 200 mL of liquid medium with 10 g/L of glucose, 200 g/L of potato, and 100 mL of leach liquor of soil per liter at 30 °C and 120 rpm for four days. The broth was centrifuged (5000 rpm), and the supernatant was filtered using 0.25 µm filtration membrane to prepare cell-free culture fluids. After concentration, the remaining cell-free culture fluids (3 mL) were coated on three SD plates, on which *F*. *oxysporum* was monocultured to investigate its effect on the growth of *F*. *oxysporum*.

### Effect of HA on *F*. *oxysporum*

The pot experiment was conducted to validate the effect of HA on *F*. *oxysporum* growth. Seedlings of cut chrysanthemum were planted in 40 pots each containing 1.5 L of sterile soil (sterilized at 121 °C for 20 min) from an abandoned farmland, in which no HA was detected. Three quarters of pots were divided into three groups, and the HA contents of the groups were correspondingly adjusted to 2, 4, and 6 mg/g through the addition of HA to soil. All the pots were incubated at 23 °C–28 °C and 80–85% humidity in a greenhouse. After 10 days of cultivation, *F*. *oxysporum* suspension (50 mL) with the concentration of 4.9 g/L was inoculated into pots. The subsequent experiment was conducted for 50 days, and the quantity of *F*. *oxysporum* in soil was counted on days 0, 20, and 40 using the traditional method on PPA (15.0 g/L peptone, 1.0 g/L K_2_PO_4_, 0.5 g/L MgSO_4_.7H_2_O, 1.0 g/L PCNB, 20.0 g/L agar) and PDA plate^40–42^.

### Analytical methods

The quantification of *P*. *chrysosporium* was determined with the colony forming unit (cfu) method^24,43^. After coating of the diluted soil samples on PDA plates, the plates were incubated at 35 °C for two days. The characteristic powdery white colonies were enumerated, and the suspected colonies were further identified according to the fungi identification manual^44^. Phenolic acids, including HA, VA, SA, and FA, were determined by high-performance liquid chromatography (Waters2695, Milford, MA, USA) equipped with a photodiode array detector. A linear gradient flow was used at a flow rate of 0.5 mL/min, and treatment of soil samples for phenolic acid determination was based on the method of Heimler and Pieroni^45^. The activities of urease, sucrase, and catalase in the rhizosphere were measured via phenol sodium colorimetry, 3,5-dinitrosalicylic acid colorimetry, and KMnO_4_ titration method, respectively^46^. Physiological indices, including plant height, stem diameter, leaf length and width, and chlorophyll content, were documented on days 30, 60, 90, and 120, respectively. Each time, 10 plants were randomly selected in every row, and the third leaf from the apex of the plant was used to determine chlorophyll with a SPAD-502 chlorophyll meter (Minolta, Japan). Wilt severity was also evaluated, and all the plants were ranked into five levels: 1, healthy; 2, one-fourth of the leaves turned yellow and fell; 3, one-third of the leaves turned yellow and fell; 4, one-half of the leaves turned yellow and fell; and 5, completely dead. The incidence and disease index were calculated according to the formula^47^

1 $$\text{Incidence}( \% )=\tfrac{{\rm{number}}\,{\rm{of}}\,{\rm{diseased}}\,{\rm{plants}}}{{\rm{total}}\,{\rm{number}}\,{\rm{of}}\,{\rm{investigated}}\,{\rm{plants}}}\times 100$$

2 $${\rm{Disease}}\,{\rm{index}}=\sum \tfrac{{\rm{number}}\,{\rm{of}}\,{\rm{diseased}}\,{\rm{plants}}\times {\rm{corresponding}}\,{\rm{level}}}{{\rm{total}}\,{\rm{number}}\,{\rm{of}}\,{\rm{investigated}}\,{\rm{plants}}}\times 100$$

Statistical analysis of data was carried out in the software SPSS (version 11.5), and the significance of data difference was considered on the basis of the analysis of the least significant difference (LSD) test at the level of *p* = 0.05. Dissimilarity comparison tests between the different groups were performed in R^48^ on the basis of the Bray–Curtis dissimilarity index using analysis of similarities^49^. PCA was performed to compare the microbial community profiles of the different samples following the method described by Park *et al*.^50^.

## Electronic supplementary material

Supplementary materials
