# Supplementary material for: Possible mechanisms of control of Fusarium wilt of cut chrysanthemum by Phanerochaete chrysosporium in continuous cropping fields: A case study
Source: Sci Rep. 2017 Nov 22;7:15994. doi: 10.1038/s41598-017-16125-7 (PMC5700048; doi:10.1038/s41598-017-16125-7)
Supplement: Supplementary file 1 — Supplementary materials [file 41598_2017_16125_MOESM1_ESM.doc]

**Possible mechanisms of control of Fusarium wilt of cut chrysanthemum by *Phanerochaete chrysosporium* in continuous cropping fields: A case study**

Li Ping^1※^, Chen Jingchao^1^, Li Yi ^1※^, Zhang Kun^1^, Wang Hailei ^1, 2^ *

1, Henan Province Engineering Laboratory for Bioconversion Technology of Functional Microbes, College of Life Sciences, Henan Normal University, Xinxiang 453007, China

2, Advanced Environmental Biotechnology Center, Nanyang Environment and Water Research Institute, Nanyang Technological University, Singapore 637141, Singapore

^※^These authors contributed equally to this work.

* Corresponding author: Wang Hailei (E-mail: whl@ htu.cn)


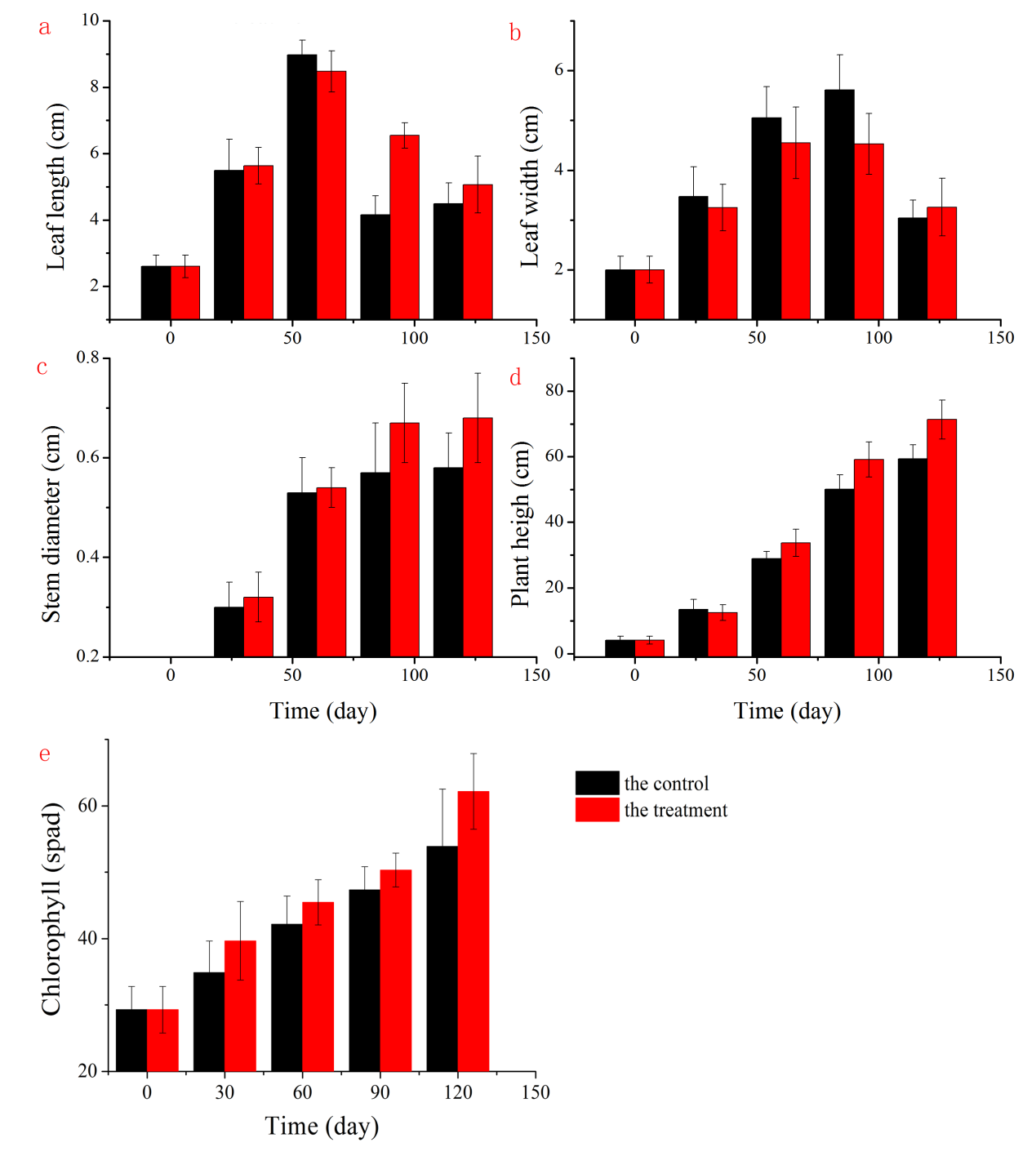


Fig.S1 The growth indice of cut chrysanthemum in the control and treatment groups after inoculation of *P. chrysosporium*. (a) leaf length; (b) leaf width; (c)stem diameter; (d) plant height and (e) chlorophyll. Mean values±standard errors (SD) are given (n = 10).


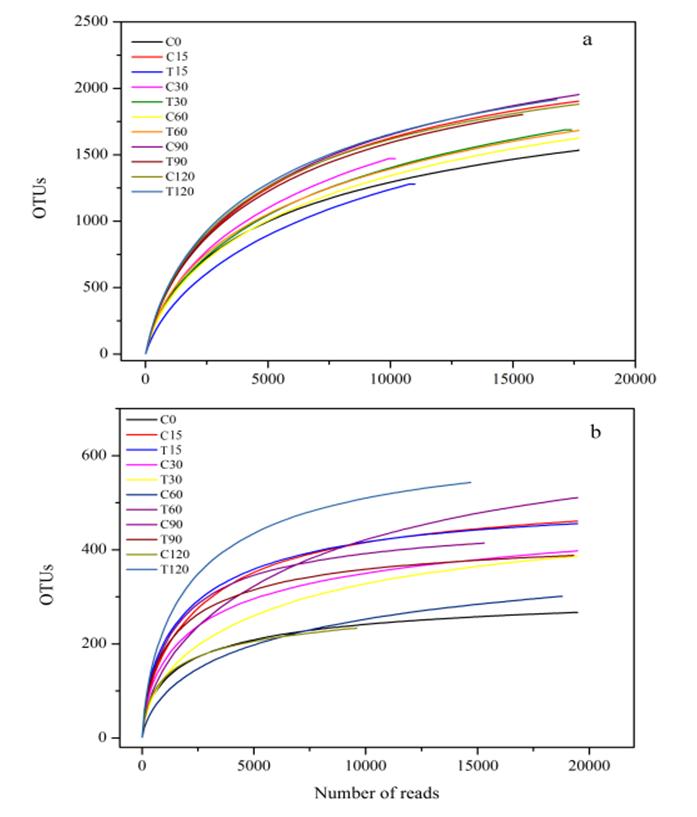


Fig.S2 Rarefaction analysis of the pyrosequencing reads of bacteria and eukaryotic cells across sludge samples at the 97% sequence similarity. (a) bacteria; (b) eukaryotic cells.


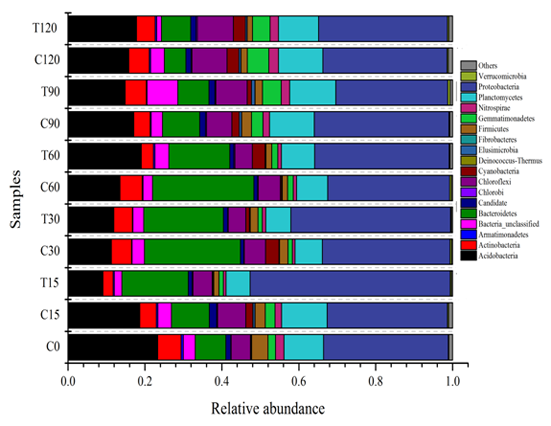


Fig.S3 Bacterial community structures in soil samples at the phylum level.


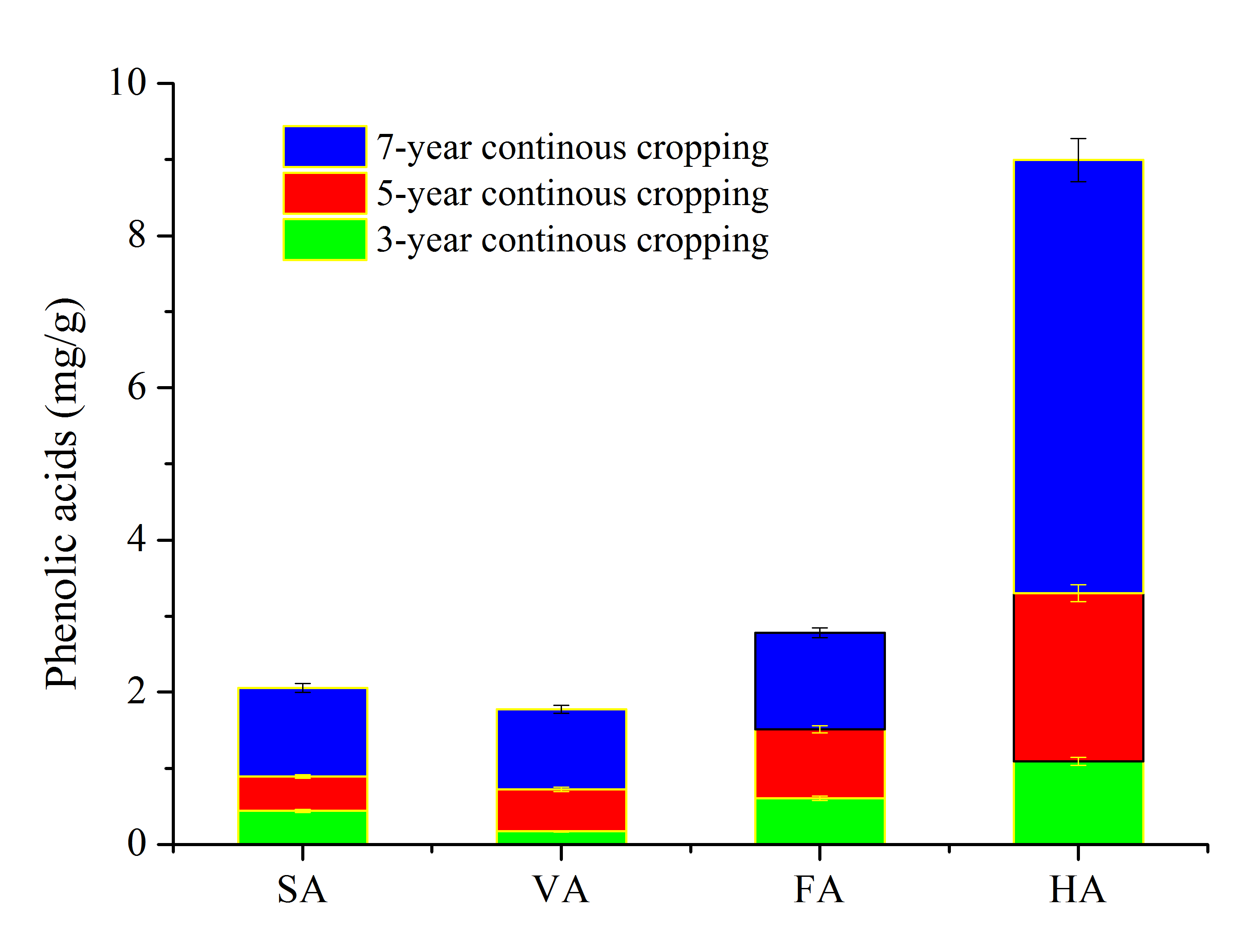


Fig.S4 The contents of syringic acid (SA), vanillic acid (VA), ferulic acid (FA) and p-hydroxybenzoic acid (HA) in soils with different planting years of cut chrysanthemum. Mean values±SD are given (n = 10).
